# Association between neutrophil-percentage-to-albumin ratio and periodontitis: insights from a population-based study

**DOI:** 10.3389/fnut.2025.1551349

**Published:** 2025-04-11

**Authors:** Ziyang Zheng, Xinyu Xie, Lan Wang, Mingzhang Xu, Jiaqi He, Yunyi Deng, Ke Yu

**Affiliations:** ^1^Department of Oral Implantology, The Affiliated Stomatological Hospital, Southwest Medical University, Luzhou, China; ^2^Luzhou Key Laboratory of Oral and Maxillofacial Reconstruction and Regeneration, Luzhou, China; ^3^Institute of Stomatology, Southwest Medical University, Luzhou, China

**Keywords:** NPAR, NHANES, epidemiology, periodontitis, inflammation

## Abstract

**Background:**

Periodontal diseases, characterized by the loss of tooth-supporting structures, are highly prevalent in the general population. The Neutrophil-Percentage-To-Albumin Ratio (NPAR) has been identified as a promising biomarker for systemic inflammation, but its relationship with periodontal disease has not been thoroughly investigated. Despite growing interest in its role in other chronic conditions, the specific connection between NPAR and periodontal disease remains underexplored and requires further examination to understand its potential clinical applications.

**Methods:**

A population-based analysis was performed using data from the National Health and Nutrition Examination Survey (NHANES), with a total of 8,389 participants included with complete full-mouth periodontal examination, NPAR related index and covariates. NPAR was employed as the primary independent variable, the periodontitis and clinical periodontal parameters were set to the outcomes along with tooth counts and functional dentition as the sensitivity outcomes. To investigate its association between NPAR and periodontitis, weighted multivariate linear and logistic regression analyses were conducted. Sensitivity and replication analyses were also carried out to assess the robustness and reliability of the findings.

**Results:**

This population-based study revealed a significant association between elevated NPAR levels and a higher likelihood of periodontitis, increased attachment loss (AL), and probing depth (PD). After full adjustment for potential confounders, NPAR was significantly associated with periodontitis (OR = 1.04, *p* = 0.005), attachment loss (β = 0.03, *p* < 0.001), and probing depth (β = 0.02, *p* < 0.001). Furthermore, the highest quartile of NPAR remained significantly associated with periodontitis (OR = 1.34, *p* = 0.010), AL (β = 0.15, *p* < 0.001) and PD (β = 0.09, *p* < 0.001). A significant trend was observed, with periodontitis strongly associated with increasing NPAR levels. These findings were further validated by the sensitivity analyses with decreased tooth counts (β = −0.08, *p* < 0.005) and the lower incidence of functional dentition (OR = 0.96, *p* = 0.030). Additionally, the replication analysis also enhanced the roundness of the results (OR = 1.07, *p* < 0.001).

**Conclusion:**

This population-based study demonstrated a statistically significant positive relationship between NPAR and the prevalence of periodontitis, NPAR has been recognized as a potential biomarker for periodontal disease. Additional longitudinal research are needed to confirm these findings and investigate the clinical implications of NPAR in managing periodontal conditions.

## Introduction

Periodontitis has traditionally been regarded as an infectious disease caused by bacteria, however, bacteria alone are insufficient to initiate and progress the disease (1). In the development of periodontitis, a key factor is low-grade inflammation (LGI), which leads to the activation of immune responses and the inflammatory phenotype associated with the disease. This chronic, mild inflammation contributes to the progressive damage of the tooth-supporting structures, promoting tissue breakdown, which also serve as key factors for many chronic diseases (2). LGI links periodontitis to cardiovascular diseases, respiratory diseases, diabetes, and neuroinflammatory conditions (3, 4). During the period from 2011 to 2020, among dentate adults the prevalence of periodontitis was estimated to be around 62%, approximately 23.6% were affected by severe cases of the disease among these individuals (5). However, the true prevalence could surpass 50%, as previous estimates have likely been too low (6).

Currently, due to a lack of awareness of early symptoms, periodontitis is often diagnosed at a later stage, leading to tooth loosening and loss, which considerably diminishes the quality of life for patients and creates a significant financial burden (7, 8). An urgent need exists to develop effective biomarkers for the evaluation, diagnosis, and management of periodontitis. Blood tests and physical examinations are routine parts of regular health checkups. Utilizing biomarkers from routine physical exams for disease prevention and treatment, including periodontitis, is essential (9).

Chronic periodontitis, characterized by elevated inflammatory cytokines, is associated with systemic conditions such as cardiovascular disease, diabetes, and cancer, suggesting a two-way relationship between oral and systemic inflammation (10). Furthermore, neutrophils, previously viewed as simple immune responders, are now recognized for their complex roles in both acute and chronic inflammation, adaptive immunity, and tissue-specific immune responses (11). Moreover, albumin levels showed an inverse correlation with C-reactive protein (CRP) and white blood cell counts, both of which are markers of inflammation. In contrast, positive correlations were identified between albumin levels and platelets, as well as with hemoglobin level (12). Common blood count results include increased white blood cell and neutrophil levels, elevated erythrocyte sedimentation rate, and decreased mean platelet volumes in patients with periodontitis (13). Additionally, serum albumin concentration was found to be inversely related to the presence of periodontal disease in elderly subject and patients who were receiving chronic outpatient hemodialysis (14, 15). The Neutrophil Percentage-to-Albumin Ratio (NPAR) combines the levels of neutrophils and albumin, two distinct biomarkers that, respectively, reflect acute and chronic inflammation, offering a new measure of the inflammatory status (16). In adults with chronic obstructive pulmonary disease, an elevated NPAR is linked to increased mortality risks and demonstrates superior performance compared to other inflammatory biomarkers in predicting 5-year all-cause mortality (17), offering a more comprehensive assessment of conditions such as liver disease, chronic kidney disease, depression, metabolic syndrome, and diabetic retinopathy (18–21). Moreover, its predictive value for mortality has been established in patients with myocardial infarction, chronic heart failure, atrial fibrillation, and sepsis (16, 22–24).

While the relationship between inflammation and periodontal disease has been extensively explored, to the best of our knowledge, no studies have thoroughly investigated the association between the novel biomarker NPAR and periodontitis. As a new biomarker underlining the inflammatory response, NPAR has not yet been extensively studied or widely applied in periodontal research, limiting our understanding of its potential role in diagnosing and monitoring periodontal diseases. It is proposed that NPAR could act as a novel biomarker for the prevention, diagnosis, treatment, and management of periodontal diseases. The aim of this population-based study is to investigate the relationship between NPAR and periodontitis within a large, nationally representative population, providing valuable insights into the potential role of this novel biomarker in understanding and managing periodontal disease.

## Materials and methods

### Study design

The National Health and Nutrition Examination Survey (NHANES) is a large-scale, multistage public health survey designed to represent the noninstitutionalized population of the United States. Managed by the Centers for Disease Control and Prevention (CDC) and the National Center for Health Statistics (NCHS), NHANES provides a wealth of nationally representative data on a wide range of health and nutrition indicators. Furthermore, the NHANES protocol undergoes regular review and approval by the NCHS Ethics Review Committee, ensuring adherence to ethical research standards.

**Figure 1:**
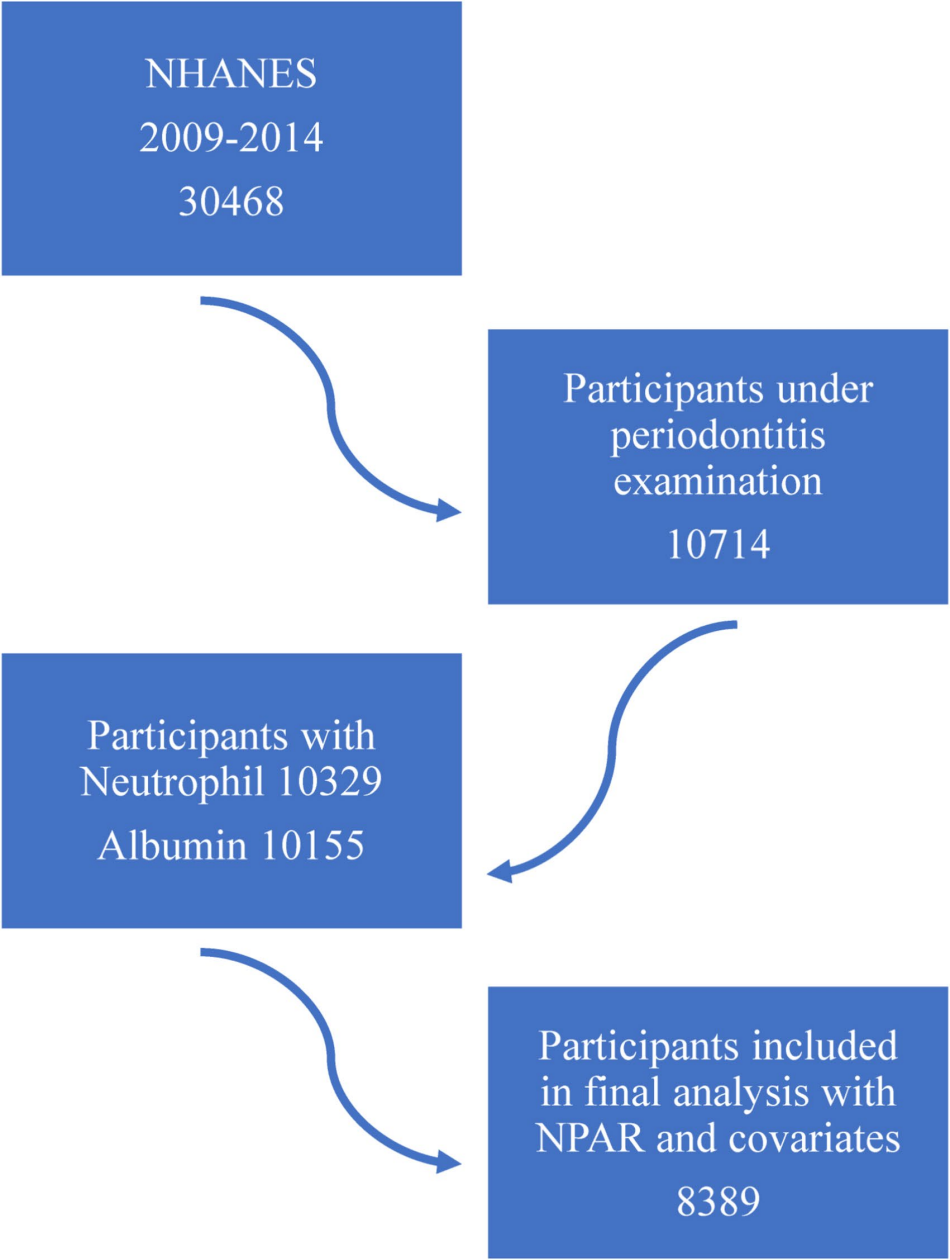
The flowchart of this study.

This population-based study is from 2009 to 2014 NHANES, based on three cycles, and includes participants who underwent periodontal examinations and complete NAPR-related data. A total of 30,648 individuals were surveyed from 2009 to 2014, with 10,714 undergoing periodontal examinations, 10,329 having blood cell examinations, and 10,155 undergoing albumin examinations. After excluding individuals with incomplete full-mouth periodontal examinations, missing blood biochemical tests, and unavailable covariate data, the final sample consisted of 8,389 participants. Participant inclusion is shown in Figure 1.

### The definition of NPAR

The neutrophil percentage was measured using the Beckman Coulter DxH 900 Automated Hematology Analyzer (Beckman Coulter, Brea, CA, United States), a precise device which quantifies the absolute number of neutrophils as part of the total white blood cell count. The analyzer does not perform any density-based separation of neutrophils, nor does it distinguish between high or low-density neutrophils. Additionally, albumin concentration was measured using the Beckman UniCel^®^ DxC 660i Synchron Access which quantifies albumin levels in serum samples through a biochemical method. The NPAR was determined using the following formula: the neutrophil percentage (calculated as a proportion of the total white blood cell count) multiplied by 100, then divided by the albumin concentration (in g/dL) (25).

### The definition of periodontitis

For periodontitis, the definition and severity of periodontitis are according to the 2017 World Workshop on the Classification of Periodontal and Peri-Implant Diseases and Conditions (26). However, due to the absence of data on factors such as furcation involvement and tooth loss etiology in the NHANES database, a 4 mm interdental clinical attachment loss threshold was set to align with periodontitis prevalence as defined by the CDC/AAP criteria. The presence or absence of functional and non-functional dentition was determined by the number of natural teeth possessed by each participant, with the exception of primary teeth (27). Detailed definitions are provided in Table 1.

**Table 1:** The case, definition and data sources of this study.

Variable	Definition
Cross-sectional study	
NPAR	Neutrophil-percentage-to-albumin ratio by hematology and biochemistry
Periodontitis	Interdental clinical attachment loss (CAL) detectable at ≥4 non-adjacent natural teeth, or buccal/oral CAL ≥3 mm with concomitant pocketing >3 mm at ≥2 natural teeth
Functional dentition	Having ≥20 natural permanent teeth independent of their condition or position
Non-functional dentition	Having ≤19 natural permanent teeth independent of their condition or position

AL, Attachment loss; BMI, Body mass index; NPAR, Neutrophil-percentage-to-albumin ratio; PD, Probing depth.

### Assessment of covariates

This study incorporated both categorical and continuous covariates according to previous study (28). Demographic factors included in the study were gender (male or female), race/ethnicity (Mexican American, non-Hispanic White, non-Hispanic Black, and other), education level (below high school or high school and above), and marital status (married, living with a partner, widowed, divorced, separated, or never married), smoking status (never, former, or current), and poverty income ratio (PIR), while clinical and health-related factors encompassed diabetes mellitus (determined by fasting plasma glucose ≥7.0 mmol/L or hemoglobin A1c (HbA1c) ≥6.5%, taking hypoglycemic drugs, or self-reported diabetes previously diagnosed by a physician), hypertension (determined by as taking antihypertensive medications, having a systolic blood pressure (BP) ≥140 mm Hg or a diastolic BP ≥90 mm Hg, or prior diagnosis of hypertension by a physician), coronary heart disease (CHD determined by the question: “Have you ever been told you had coronary heart disease” with a “Yes” answer), angina (determined by the question: “Have you ever been told you had angina/angina pectoris” with a “Yes” answer), heart attack (determined by the question: “Have you ever been told you had heart attack” with a “Yes” answer), chronic heart failure (CHF determined by the question: “Have you ever been told you had congestive heart failure” with a “Yes” answer), and the aforementioned four diseases were collectively defined as heart disease (any one of which could be confirmed with a “Yes” answer to the relevant question). Continuous variables such as age, mean AL and PD, tooth counts, neutrophil percent, serum albumin, NPAR values, and body mass index (BMI). The definition of certain covariates is detailed in Supplementary Table S1.

### Statistical analysis

Continuous variables were expressed as the mean with standard deviation (SD), whereas categorical variables were represented as frequencies (n) and percentages (%). Weighted Student’s *t*-tests were employed for the comparison of continuous variables, while Chi-square tests were used to analyze categorical variables. Multivariate logistic and linear regression models were utilized to assess the impact of NPAR on periodontitis across three different models. The results were reported using the odds ratio (OR), β-coefficient, and confidence interval (CI) to quantify the strength and direction of the associations, providing a comprehensive understanding of the relationship between NPAR and periodontal parameters: Model I (no adjustment), Model II (adjustment for gender, age, race, socioeconomic status, and education), and Model III (adjustment for all relevant covariates) were used to examine the relationships between the variables under consideration. Subgroup analyses were conducted on age, sex, education level, poverty, smoking, alcohol consumption, BMI, diabetes, hypertension, marital status, and heart disease to explore the relationship between NPAR and periodontitis in specific groups.

Sensitivity analyses were carried out to examine the associations between NPAR and tooth counts, as well as functional dentition. Furthermore, a replication analysis using NHANES 1999–2004 data was conducted to enhance the reliability of the findings, focusing on the association between NPAR and periodontitis across different years. Additionally, restricted cubic spline (RCS) analysis was performed to investigate the non-linear relationship between NPAR and periodontitis. All analyses were performed using R software (version 4.3.3), incorporating the survey design and weighting variables. A *p*-value of less than 0.05 was considered indicative of statistical significance.

## Results

### Baseline characteristics of participants

Details of the inclusion process is shown in Figure 1. The basic characteristics of inclusion participants is shown in Table 2. This study included 8,389 individuals, with 4,069 non-periodontitis and 4,320 periodontitis cases. Among the participants, 49.6% were female and 50.4% were male. Significant differences were observed across various NPAR levels in several factors, including periodontal parameters (AL, PD and tooth count), neutrophil and serum albumin levels, age, sex, race, marital status, poverty levels, smoking, alcohol consumption, diabetes, hypertension, heart disease, and functional dentition status. The basic information of NPAR is presented in Supplementary Figure S1.

**Table 2:** Baseline characteristics stratified by NPAR quartiles in this study.

Variable	Total	Q1	Q2	Q3	Q4	*P*-value
Age, (years)	51.08 (0.25)	49.00 (0.38)	49.87 (0.37)	52.12 (0.46)	53.29 (0.37)	<0.0001
Mean AL, (mm)	1.64 (0.03)	1.55 (0.03)	1.59 (0.03)	1.66 (0.04)	1.76 (0.04)	<0.0001
Mean PD, (mm)	1.43 (0.02)	1.40 (0.03)	1.40 (0.02)	1.43 (0.02)	1.48 (0.02)	0.003
Tooth counts	24.12 (0.12)	24.58 (0.14)	24.52 (0.15)	24.11 (0.19)	23.26 (0.21)	<0.0001
Neutrophil percentage, %	58.71 (0.17)	47.74 (0.20)	56.38 (0.13)	61.84 (0.15)	68.44 (0.15)	<0.0001
Serum albumin, (g/dl)	4.28 (0.01)	4.43 (0.01)	4.37 (0.01)	4.27 (0.01)	4.06 (0.01)	<0.0001
NPAR	13.80 (0.05)	10.79 (0.04)	12.92 (0.02)	14.49 (0.01)	16.91 (0.04)	<0.0001
**Sex, *n* (%)**						<0.0001
Female	4,149 (49.58)	910 (44.06)	987 (45.95)	1,061 (49.66)	1,191 (58.75)	
Male	4,240 (50.42)	1,189 (55.94)	1,108 (54.05)	1,038 (50.34)	905 (41.25)	
**Race, *n* (%)**						<0.0001
Mexican American	1,134 (7.40)	230 (7.32)	314 (8.15)	294 (6.86)	296 (7.25)	
Non-Hispanic Black	1,656 (9.67)	609 (14.81)	359 (8.15)	321 (7.57)	367 (8.63)	
Non-Hispanic White	3,880 (71.58)	783 (64.98)	973 (72.22)	1,054 (74.47)	1,070 (74.14)	
Other Hispanic	796 (4.93)	200 (5.51)	203 (4.78)	203 (4.77)	190 (4.70)	
Other race-including multi-racial	923 (6.42)	277 (7.38)	246 (6.70)	227 (6.33)	173 (5.28)	
**Education level, *n* (%)**						0.43
Below high school	1,790 (13.81)	436 (13.86)	443 (13.40)	423 (12.77)	488 (15.31)	
High school	1,826 (20.85)	439 (20.13)	451 (20.48)	477 (21.11)	459 (21.67)	
Above high school	4,773 (65.34)	1,224 (66.01)	1,201 (66.12)	1,199 (66.12)	1,149 (63.02)	
**Poverty, *n* (%)**						0.02
Low income	2,400 (17.77)	570 (17.35)	586 (17.85)	604 (17.14)	640 (18.76)	
Moderate income	2,986 (34.22)	736 (32.38)	720 (32.12)	751 (35.26)	779 (37.14)	
High income	3,003 (48.01)	793 (50.27)	789 (50.03)	744 (47.60)	677 (44.10)	
**Marital status, *n* (%)**						<0.0001
Married/living with partner	5,455 (69.77)	1,392 (72.04)	1,404 (71.73)	1,407 (71.62)	1,252 (63.61)	
Divorced/single/living alone	2,931 (30.19)	706 (27.96)	691 (28.27)	690 (28.38)	844 (36.39)	
**BMI, *n* (%)**						<0.0001
Normal	2,201 (26.31)	614 (29.77)	596 (29.52)	523 (23.85)	468 (22.12)	
Obese	3,294 (38.08)	679 (29.58)	738 (33.66)	856 (40.23)	1,021 (48.75)	
Overweight	2,894 (35.61)	806 (40.65)	761 (36.82)	720 (35.92)	607 (29.13)	
**Alcohol consumption, *n* (%)**						<0.0001
Never	1,109 (9.98)	290 (10.25)	264 (8.96)	255 (9.22)	300 (11.66)	
Former	1,433 (14.37)	304 (11.27)	338 (13.08)	358 (14.59)	433 (18.52)	
Mild	3,053 (39.66)	774 (39.78)	786 (43.30)	781 (40.47)	712 (34.72)	
Moderate	1,283 (17.89)	348 (20.85)	308 (16.85)	329 (17.52)	298 (16.57)	
Heavy	1,511 (18.09)	383 (17.85)	399 (17.81)	376 (18.20)	353 (18.52)	
**Smoke, *n* (%)**						<0.001
Never	4,656 (55.87)	1,184 (56.30)	1,238 (59.14)	1,160 (55.89)	1,074 (51.90)	
Former	2,170 (26.87)	557 (28.74)	503 (25.24)	530 (25.23)	580 (28.59)	
Now	1,563 (17.26)	358 (14.96)	354 (15.62)	409 (18.88)	442 (19.51)	
**Diabetes, *n* (%)**						<0.0001
No	7,009 (87.41)	1,818 (91.24)	1,807 (91.38)	1,755 (85.40)	1,629 (81.59)	
Yes	1,380 (12.59)	281 (8.76)	288 (8.62)	344 (14.60)	467 (18.41)	
**Hypertension, *n* (%)**						<0.0001
No	4,664 (59.38)	1,229 (63.68)	1,220 (62.82)	1,178 (58.04)	1,037 (52.97)	
Yes	3,725 (40.62)	870 (36.32)	875 (37.18)	921 (41.96)	1,059 (47.03)	
**Heart disease, *n* (%)**						<0.001
No	7,849 (94.15)	2,001 (95.66)	1,987 (95.26)	1,958 (94.04)	1,903 (91.60)	
Yes	540 (5.85)	98 (4.34)	108 (4.74)	141 (5.96)	193 (8.40)	
**Functional dentition, *n* (%)**						<0.0001
Non-function	1,719 (14.20)	386 (11.78)	381 (12.54)	418 (14.53)	534 (17.97)	
Function	6,670 (85.80)	1,713 (88.22)	1,714 (87.46)	1,681 (85.47)	1,562 (82.03)	
**Periodontitis**						<0.0001
No	4,069 (57.47)	1,040 (60.59)	1,085 (61.12)	1,039 (56.61)	905 (51.44)	
Yes	4,320 (42.53)	1,059 (39.41)	1,010 (38.88)	1,060 (43.39)	1,191 (48.56)	

Continuous variables were showed as mean ± (SD), categorical variables were showed as *n* (%). AL, attachment loss; BMI, Body mass index; NPAR, Neutrophil-percentage-to-albumin ratio; PD, probing depth.

### Correlation between NPAR levels and periodontitis

As illustrated in Table 3, a detailed relationship is presented between NPAR quartiles and periodontitis. Multivariate logistic and linear regression in model III demonstrated a correlation between NPAR levels and higher incidence of periodontitis [OR (CI): 1.04 (1.01, 1.07), *p* = 0.005], increased mean attachment loss [β (CI): 0.03 (0.02, 0.04), *p* < 0.001], and elevated mean probing depth [β (CI): 0.02 (0.01, 0.02), *p* < 0.001] when NPAR treated as a continuous variable.

**Table 3:** Association between NPAR and periodontitis.

Variables		Model I		Model II		Model III	
Continuous	Periodontitis	OR[CI]	*P*	OR[CI]	*P*	OR[CI]	*P*
		1.06 (1.03, 1.09)	<0.001	1.06 (1.03, 1.09)	<0.001	1.04 (1.01, 1.07)	0.005
**Categorical**							
Q1	Reference						
Q2		0.98 (0.86, 1.11)	0.978	1.01 (0.87, 1.16)	0.920	1.00 (0.87, 1.16)	0.950
Q3		1.18 (1.00, 1.39)	0.049	1.18 (1.00, 1.40)	0.049	1.12 (0.95, 1.31)	0.160
Q4		1.45 (1.21, 1.74)	<0.001	1.48 (1.22, 1.78)	<0.001	1.34 (1.09, 1.63)	0.010
	*P* for trend		0.004		<0.001		0.001
Continuous	AL	β[CI]		β[CI]		β[CI]	
		0.03 (0.02, 0.05)	<0.001	0.03 (0.02, 0.04)	<0.001	0.03 (0.02, 0.04)	<0.001
**Categorical**							
Q1	Reference						
Q2		0.03 (−0.03, 0.09)	0.280	0.05 (0.00, 0.10)	0.060	0.05 (0.00, 0.11)	0.060
Q3		0.10 (0.03, 0.18)	0.010	0.10 (0.02, 0.17)	0.010	0.07 (0.00, 0.14)	0.040
Q4		0.21 (0.13, 0.29)	<0.001	0.19 (0.12, 0.26)	<0.001	0.15 (0.08, 0.22)	<0.001
	*P* for trend		<0.001		<0.001		<0.001
Continuous	PD	β[CI]		β[CI]		β[CI]	
		0.01 (0.01, 0.02)	0.001	0.02 (0.01, 0.03)	<0.001	0.02 (0.01, 0.02)	<0.001
**Categorical**							
Q1	Reference						
Q2		0 (−0.03, 0.04)	0.960	0.02 (−0.01, 0.05)	0.210	0.02 (−0.01, 0.05)	0.280
Q3		0.04 (0.00, 0.08)	0.060	0.07 (0.03, 0.10)	<0.001	0.05 (0.01, 0.08)	0.010
Q4		0.09 (0.03, 0.14)	0.002	0.12 (0.07, 0.16)	<0.001	0.09 (0.05, 0.14)	<0.001
	*P* for trend		<0.001		<0.001		<0.001

AL: attachment loss; NPAR, Neutrophil-percentage-to-albumin ratio; PD, probing depth.

Weighted multivariate linear and logistic regression analyses were performed with NPAR as the main independent variable, and β-coefficients and odds ratios (OR) were calculated to assess the association between NPAR and periodontal diseases in Model I (unadjusted), Model II (adjusted for age, race, gender, poverty level, education and marital status), and Model III (fully adjusted for all covariates).

When NPAR was divided into four quartiles, multivariate logistic and linear regression in model III revealed that the highest quartile was positively correlated with higher prevalence of periodontitis [OR (CI): 1.34 (1.09, 1.63), *p* = 0.010], increased mean attachment loss [β (CI): 0.15 (0.08, 0.22), *p* < 0.001], and elevated mean probing depth [β (CI): 0.09 (0.05, 0.14), *p* < 0.001], compared to the lowest NPAR quartile according to model III. Furthermore, there was a significance association between NPAR levels and the periodontitis and relative clinical parameters (*P* for trend = 0.001, <0.001, and <0.001 for periodontitis, AL, and PD respectively).

### Sensitivity analysis

As shown in Table 4, in the associations between NPAR and tooth counts and functional dentition status, a robust positive correlation was also established. In the NPAR impact on the tooth counts, each unit increase in the NPAR resulted in a decrease of 0.08 units in tooth counts [β (CI): −0.08 (−0.14, −0.03), *p* = 0.005], and participants in the highest quartile showed a 0.44-unit decrease compared to the lowest quartile [β (CI): −0.44(−0.81, −0.08), *p* = 0.020] according to Model III, also there is a trend significance of NPAR levels and tooth counts (*P* for trend < 0.001). The relationship was found exclusively when NPAR was considered as a continuous variable in Model III, with functional dentition status, suggesting a 4% increase in the risk of non-functional dentition [OR (CI): 0.96 (0.93, 1.00), *p* = 0.030], and there was no significant association when NPAR was treated as categorical variable. Consistent with the main findings, NHANES year from 1999 to 2004 presented in Table 5 showed that NPAR increased periodontitis prevalence [OR (CI): 1.07 (1.04, 1.10), *p* < 0.001], with the highest NPAR group showing the largest increase [OR (CI): 1.59 (1.20, 2.10), *p* = 0.005]. The sensitivity analysis conducted provided further evidence supporting the reliability and stability of the study’s findings.

**Table 4:** Association between NPAR and tooth counts, functional dentition.

Variables		Model I		Model II		Model III	
Continuous	Tooth counts	β[CI]	*P*	β[CI]	*P*	β[CI]	*P*
		0.22 (−0.29, −0.15)	<0.001	−0.14 (−0.20, −0.08)	<0.001	−0.08 (−0.14, −0.03)	0.005
**Categorical**							
Q1	Reference						
Q2		−0.05 (−0.35, 0.24)	0.710	−0.14 (−0.45, 0.16)	0.350	−0.11 (−0.45, 0.22)	0.490
Q3		−0.47 (−0.82, −0.12)	0.010	−0.26 (−0.65, 0.13)	0.180	−0.09 (−0.47, 0.30)	0.650
Q4		−1.32 (−1.72, −0.92)	<0.001	−0.80 (−1.17, −0.44)	<0.001	−0.44 (−0.81, −0.08)	0.020
	*P* for trend		<0.001		<0.001		0.036
Continuous	Functional dentition	OR[CI]		OR[CI]		OR[CI]	
		0.91 (0.89, 0.94)	<0.001	0.93 (0.90, 0.96)	<0.001	0.96 (0.93, 1.00)	0.030
**Categorical**							
Q1	Reference						
Q2		0.93 (0.78, 1.11)	0.420	0.88 (0.71, 1.07)	0.190	0.90 (0.71, 1.14)	0.380
Q3		0.79 (0.65, 0.95)	0.010	0.78 (0.62, 0.98)	0.030	0.88 (0.69, 1.13)	0.300
Q4		0.61 (0.51, 0.72)	<0.001	0.69 (0.57, 0.84)	<0.001	0.83 (0.66, 1.04)	0.110
	*P* for trend		<0.001		<0.001		0.142

Note: NPAR, Neutrophil-percentage-to-albumin ratio.

Weighted multivariate linear and logistic regression analyses were performed with NPAR as the main independent variable, and β-coefficients and odds ratios (OR) were calculated to assess the association between NPAR and tooth counts and functional dentition in Model I (unadjusted), Model II (adjusted for age, race, gender, poverty level, education and marital status), and Model III (fully adjusted for all covariates).

**Table 5:** Association between NPAR and periodontitis based on NHANES from 1999 to 2004.

Variables		Model I		Model II		Model III	
Continuous	Periodontitis	OR[CI]	*P*	OR[CI]	*P*	OR[CI]	*P*
		1.05 (1.03, 1.08)	<0.001	1.07 (1.05, 1.10)	<0.001	1.07 (1.04, 1.10)	<0.001
**Categorical**							
Q1	Reference						
Q2		1.15 (0.89, 1.48)	0.285	1.21 (0.91, 1.62)	0.190	1.25 (0.90, 1.73)	0.160
Q3		1.29 (1.00, 1.66)	0.047	1.38 (1.06, 1.79)	0.020	1.36 (1.01, 1.83)	0.040
Q4		1.57 (1.27, 1.96)	<0.001	1.68 (1.32, 2.14)	<0.001	1.59 (1.20, 2.10)	0.005
	*P* for trend		<0.001		<0.001		<0.001

Note: NPAR, Neutrophil-percentage-to-albumin ratio.

Weighted multivariate linear and logistic regression analyses were performed with NPAR as the main independent variable, and odds ratios (OR) was calculated to assess the association between NPAR and periodontitis in Model I (unadjusted), Model II (adjusted for age, race, gender, poverty level, education and marital status), and Model III (fully adjusted for all covariates).

### Subgroup analysis

**Figure 2:**
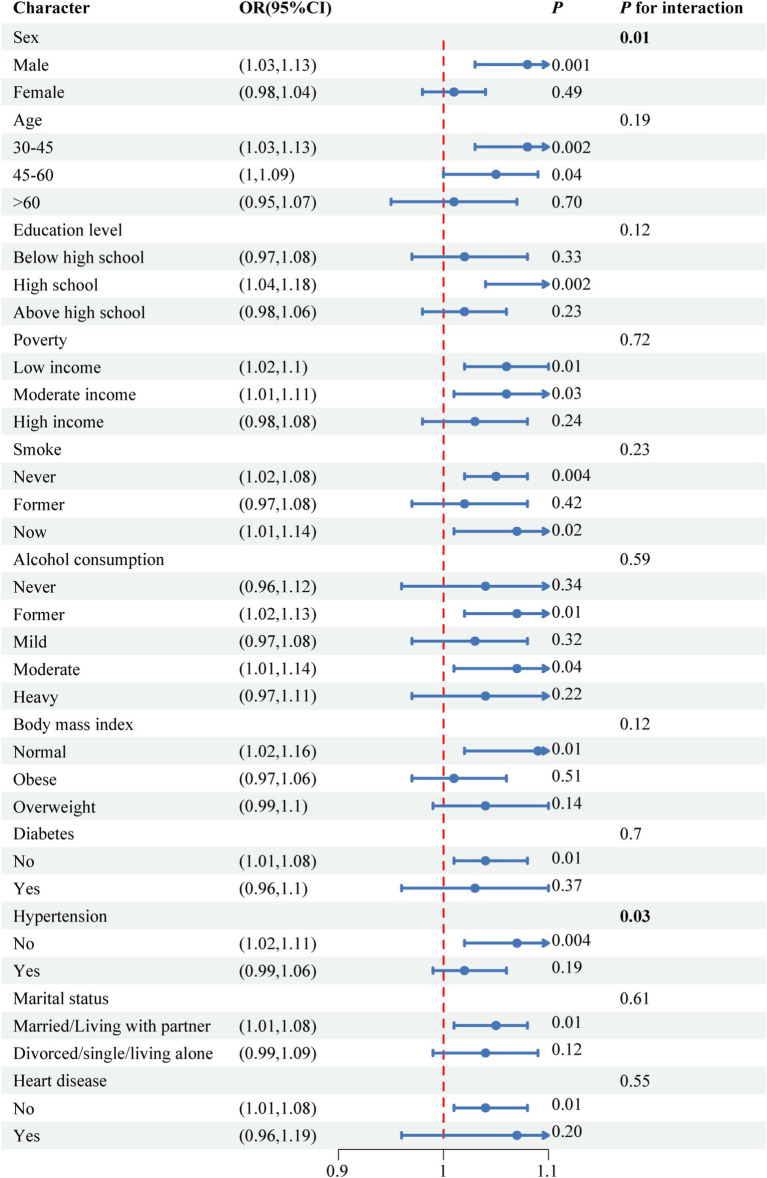
Subgroup analyses for relationship between NPAR and periodontitis.

**Figure 3:**
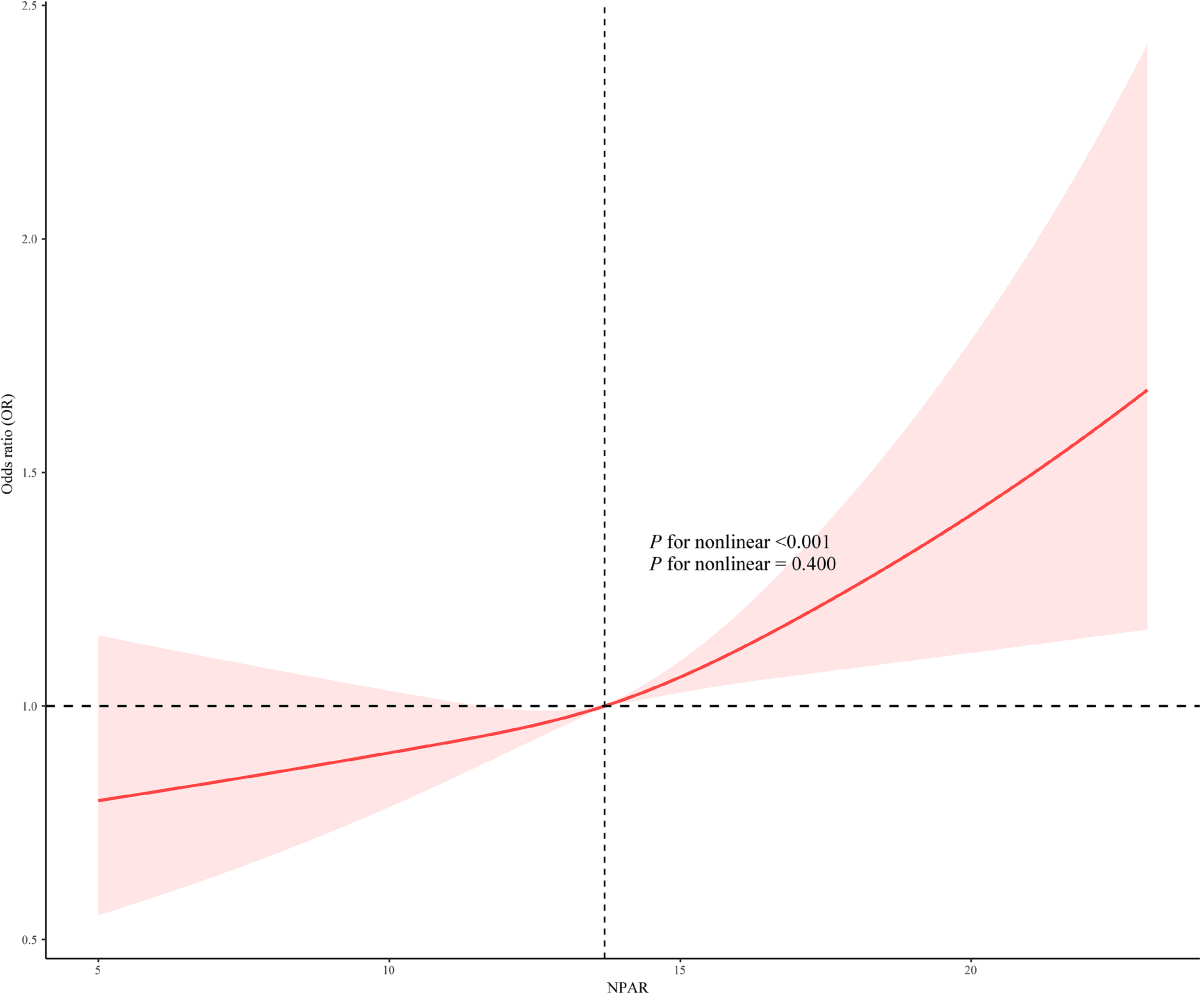
Restricted cubic splines of the association between NPAR with periodontitis.

Figure 2 illustrates the findings from the subgroup analyses including age, sex, education level, poverty, smoking, alcohol consumption, BMI, diabetes, hypertension, marital status, and heart disease, providing a detailed overview of the relationships explored. The interactions between sex, hypertension, and NPAR in relation to periodontitis were identified (*P* for interaction <0.05) and positive correlations were observed in males [OR (CI): 1.08 (1.03, 1.13), *p* = 0.001], and participants without hypertension [OR (CI): 1.07 (1.02, 1.11), *p* = 0.004]. RCS analysis demonstrated a linear relationship between NPAR and periodontitis (*P* for nonlinear = 0.400, Figure 3).

## Discussion

This study identified a significant association between NPAR and periodontitis in a population-based representative U.S. cohort of 8,389 participants, suggesting that NPAR could serve as a potential index for periodontal disease. Our findings indicate a positive relationship between NPAR and the incidence of periodontitis, with each unit increase in NPAR being significantly associated with a higher risk of developing periodontitis. To the best of our knowledge, this is the first comprehensive cross-sectional investigation to examine the possible link between NPAR and periodontitis among U.S. adults. However, considering the study design, it is not possible to draw causal conclusions from the correlation between NPAR and periodontitis.

Neutrophil homeostasis is a complex physiological system that is subject to precise regulation. This is achieved through the coordinated processes of bone marrow production, release into the bloodstream, migration to peripheral tissues, and removal of aged neutrophils. Imbalances in neutrophil activity, whether in terms of their quantity or immune function, have been demonstrated to contribute to the development of periodontitis (29, 30). Collateral tissue damage occurs when poorly regulated neutrophilic polymorphonuclear leukocytes pursue periodontal bacteria (31). Impaired neutrophil function, as observed in hereditary conditions such as Papillon-Lefèvre syndrome, results in detrimental periodontal inflammation, causing severe tissue damage and tooth loss (32, 33), neutrophil hyperactivity is equally associated with both local and systemic inflammatory-immune responses (34, 35). Meanwhile, loss of neutrophil homing can alter the composition and pathology of the oral microbiota (36). The presence of neutrophil extracellular traps (NETs) was identified in tissue samples obtained from patients suffering from periodontitis. These traps manifested as extracellular chromatin components, in conjunction with neutrophil enzymes, which were absent in samples obtained from healthy controls (37). The enhanced degradation of NETs following periodontitis treatment has been identified as a systemic biomarker, suggesting a reduction in pro-inflammatory activity (38). Numerous reports confirm that blood, tissue, and oral neutrophils exhibit heterogeneous behavior, with distinct neutrophil populations and phenotypes. The modulation of neutrophil morphology from a para-inflammatory to a pro-inflammatory state regulates the inflammatory process in periodontal disease (39). In patients diagnosed with refractory aggressive periodontitis (RAP), neutrophil function was characterized by elevated levels of phorbol 12-myristate 13-acetate-induced oxygen radical production, in comparison to both healthy controls (HC) and chronic periodontitis (CP) patients. Furthermore, RAP patients exhibited augmented phagocytic activity in comparison to the CP group (40). The proportion of CD177^+^ neutrophils in the circulation was found to be significantly elevated in the periodontitis patient group in comparison with the HC group (41). The neutrophil phenotype in peripheral blood remained viable, with a decrease in CD62L^−^ expression following treatment. Suppressive neutrophils decreased, while normal neutrophils increased after treatment (42). Furthermore, recent advances in the field have enabled the categorization of neutrophils into various subsets, including low-density (LDN), normal-density (NDN), and pro-inflammatory N1, as well as anti-inflammatory N2 subsets. Each of these subsets exhibits distinct functional roles. The expression of LDN marker genes differed significantly between active and suppressive phenotypes, with the former promoting periodontitis over the control group (43). However, the evidence for the presence of N1 or N2 phenotypes in periodontal tissues during periodontitis is currently lacking, highlighting the need for further studies (44). The recruitment and migration of specific neutrophil phenotypes may be closely regulated by the balance of the periodontal environment.

Research has demonstrated that serum albumin levels serve as a vital indicator of periodontal disease progression in elderly individuals. Conversely, periodontal disease has also been observed to influence serum albumin concentrations (14, 45). Alterations in serum albumin levels, precipitated by periodontal disease and its concomitant therapeutic interventions, have concomitantly been shown to indicate an inverse correlation between serum albumin levels and chronic periodontitis (46). Albumin, a medium-sized housekeeping protein, performs multiple functions, including osmoregulation, anti-oxidation, and anti-inflammation. It constitutes more than half of the total composition of human serum (18). Moreover, ischemia-modified albumin is a marker of systemic inflammation in periodontal disease, showing a significant decrease following non-surgical periodontal therapy (47). Smokers with generalized chronic periodontitis had significantly lower serum albumin levels than non-smokers with the same condition (48). Moreover, patients undergoing hemodialysis with periodontitis had lower serum albumin levels (15, 49). A significant positive correlation exists between hypoalbuminemia and the Oral Assessment Guide score in newly admitted patients. Specifically, a patient with hypoalbuminemia tends to have a higher Oral Assessment Guide score, reflecting poorer oral health, and is more likely to have conditions such as respiratory and digestive diseases, a lower BMI, and absence of oral feeding, which could partially be attributed to the presence of chronic periodontal diseases (50). A review focusing exclusively on epidemiological studies found that patients with periodontal disease had lower serum albumin levels compared to healthy individuals (51), which supports the findings of this study. As albumin accounts for approximately 75% of colloid osmotic pressure, a decrease in albumin levels during chronic inflammation may lead to osmotic imbalance, affecting bone metabolism and the local tissue environment in periodontal disease (52, 53). Additionally, albumin participates in redox reactions and may reduce the availability of pro-oxidants, being preferentially oxidized to protect other macromolecules. This mechanism is also associated with various cardiovascular diseases (53, 54). Oxidative stress has been identified as a pivotal pathophysiological change associated with periodontitis (55), and albumin may play a protective role in periodontal tissues by participating in redox reactions, thereby helping to prevent periodontal damage.

The potential of targeted neutrophil and albumin mechanisms, in conjunction with the utilization of biomaterials, as therapeutic interventions for periodontitis have been a subject of exploration. Neutrophils, being pivotal in the inflammatory response to periodontal infection, have the capacity to be regulated, thus facilitating control of inflammation and the promotion of healing. Albumin, recognized for its anti-inflammatory and oxidation properties, has been the subject of investigation with regard to its potential to reduce inflammation and promote tissue repair. Furthermore, the development of biomaterials to support tissue regeneration and enhance the delivery of therapeutic agents represents a promising approach to the management of periodontitis. OTU domain-containing protein 1 (OTUD1) expression has been linked to decreased inflammatory secretion, and its depletion has been associated with severe periodontitis. The negative loop forged by OTUD1 has been shown to inhibit the polarization of neutrophils with secretory phenotype (56). Gelatin methacryloyl @MP196/exos has been shown to upregulate the expression of genes associated with neutrophil apoptosis, thereby promoting this process and, consequently, inhibiting periodontal bone loss (57). In addition, it has been demonstrated that serum albumin-crosslinked nanoparticles have the capacity to rescue multiple reactive oxygen species, including superoxide anion, free hydroxyl radicals, singlet oxygen and hydrogen peroxide. Furthermore, these nanoparticles have been shown to decrease inflammation both *in vitro* and *in vivo*, thus offering a potential treatment target for periodontitis (58).

As a combined measurement of inflammatory status, NPAR, as a dual biomarker of acute and chronic inflammation, has been drawn increasing attention for the assessment of inflammation-related diseases. The underlying mechanism under the relationship in this study remains to be extrapolated, which could be attributed to the function and homeostasis of neutrophil and albumin. Aligned with those relationship between NPAR and cardiovascular, kidney, sepsis and respiratory diseases, Previous study has previously indicated a notable correlation between NPAR and suboptimal periodontal health (59), which aligns with this study’s findings. However, due to the inclusion of confounders and the extension of outcome measures (including tooth counts and functional dentition) which could offer a relatively complete summary of periodontal leading oral health, and additional verification using NHANES data from 1999 to 2004, this study provides a relatively comprehensive and robust evaluation of NPAR as a tool for the assessment of periodontal disease.

The findings of this study suggest that NPAR has the potential to serve as a valuable tool for predicting periodontal diseases. This is due to the fact that it offers a non-invasive and highly accessible method for clinical application. Nevertheless, the present study is subject to a number of limitations. Firstly, due to the cross-sectional nature of the study, it is not possible to infer causality in order to determine whether changes in NPAR precede the development of periodontal disease. Secondly, the definition of NPAR in this study relies on a single measurement, while in reality, these biomarkers fluctuate according to the body’s condition, with stable upper and lower thresholds even in healthy individuals. Thirdly, while efforts were made to include potential confounding variables, self-reported covariates and unidentified confounders may still introduce bias. Finally, it is important to note that the results of this study are based on a sample of U.S. adults. This may limit the generalizability of the findings to other populations. It is evident that further prospective, large-scale, multi-racial, and regional studies are required to validate the findings of this study and to investigate the fundamental processes.

## Conclusion

This population-based study reveals a significant association between NPAR and periodontitis, along with its clinical periodontal indices. NPAR could function as a possible biomarker for both the prevention and assessment of periodontal disease. However, this association requires further validation in large-scale prospective studies before clinical application.

## Data Availability

The findings of this study are supported by data from the NHANES (National Health and Nutrition Examination Survey). The dataset is publicly available and can be accessed directly at the following link: https://wwwn.cdc.gov/nchs/nhanes/. The original contributions presented in the study are included in the article/Supplementary material, further inquiries can be directed to the corresponding author.
