# Supplementary material for: Association between neutrophil-percentage-to-albumin ratio and periodontitis: insights from a population-based study
Source: Front Nutr. 2025 Apr 11;12:1551349. doi: 10.3389/fnut.2025.1551349 (PMC12021642; doi:10.3389/fnut.2025.1551349)
Supplement: Supplementary file 1 [file Table_1.docx]

Table S1: Definition of Covariates.

| Body mass index  (BMI) | Normal | BMI < 25 |
| --- | --- | --- |
|  | Overweight | BMI 25 to 30 |
|  | Obese | BMI > 30 |
| Poverty income ratio  (PIR) | Low income | PIR < 1.5 |
|  | Moderate income | PIR 1.5-3.5 |
|  | High income | PIR > 3.5 |
| Smoke | Never | Individuals who have not smoked 100 cigarettes in their lifetime and are currently nonsmokers |
|  | Former | Individuals who have smoked 100 cigarettes in their lifetime but are not currently smoking |
|  | Current | Individuals who are still smoking |
| Alcohol  consumption | Never | Fewer than 12 drinks in a lifetime |
|  | Former | ≥ 12 drinks in one year but no consumption in the past year, or drank ≥ 12 drinks in a lifetime but did not drink last year |
|  | Mild | 1 drink for females, 2 for men in the past year |
|  | Moderate | 2 drinks for females, 3 for men, or binge drinking 2-4 times in the past year |
|  | Heavy | 3 drinks for females, 4 for men, or binge drinking ≥ 5 times in the past year |

Figure S1: Basic characteristics of neutrophil count, neutrophil percentage, albumin and NPAR values across NPAR quartiles.


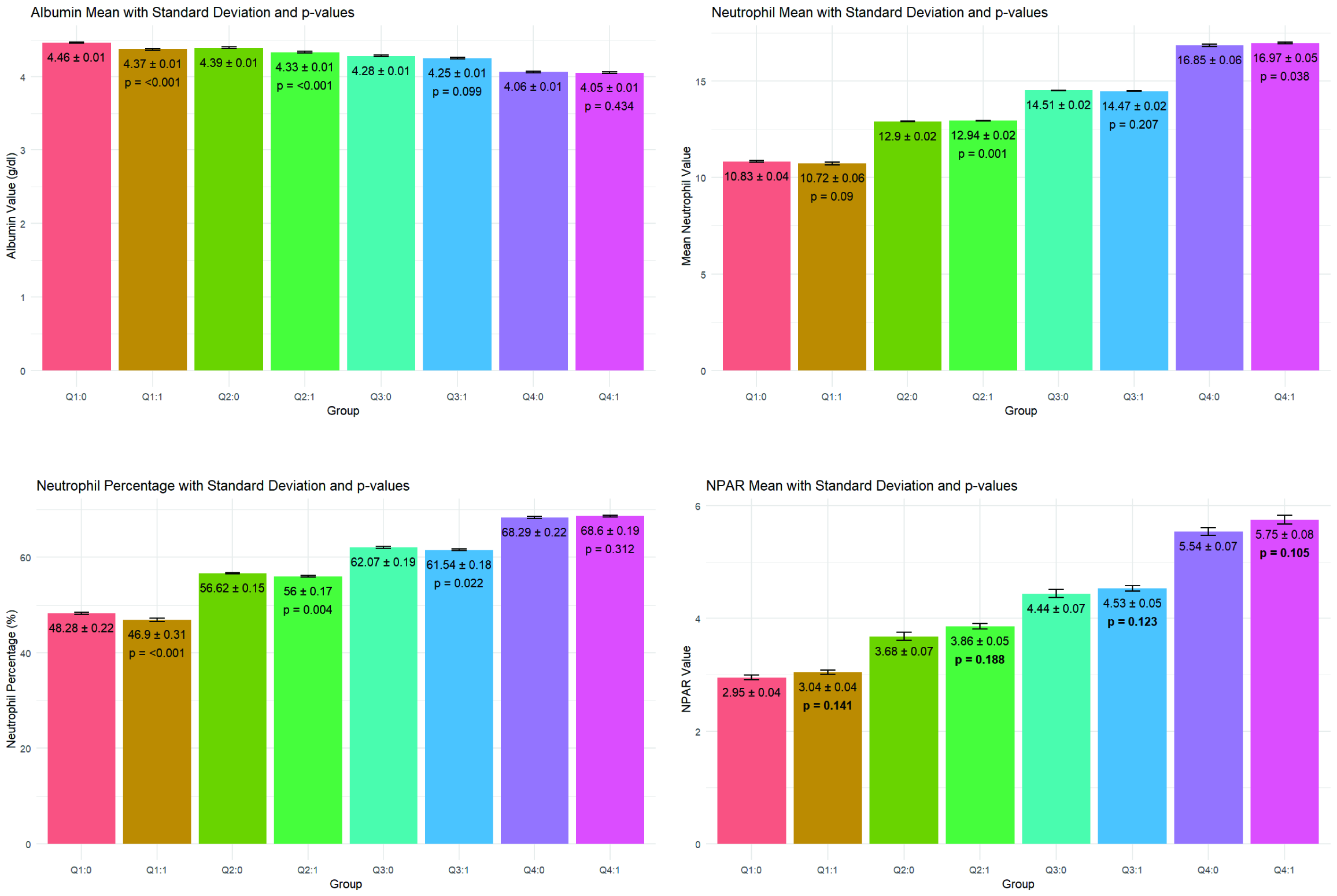


Note: NPAR: Neutrophil-Percentage-To-Albumin Ratio, 0: participants without periodontitis,1: participants with periodontitis.
